# EBV-Associated Lymphoproliferative Disorder and Hemophagocytic Lymphohistiocytosis in a Patient with Severe Celiac Disease

**DOI:** 10.1155/2018/6063519

**Published:** 2018-03-05

**Authors:** John Jacob Kinross-Wright, Kalyan Chakravarthy Potu, Brandy Pownell, Randall Lamfers, Jonathan S. Bleeker

**Affiliations:** ^1^Department of Internal Medicine, Sanford USD School of Medicine, Sioux Falls, SD, USA; ^2^Department of Pathology, Sanford USD School of Medicine, Sioux Falls, SD, USA; ^3^Division of Hematology and Oncology, Sanford USD School of Medicine, Sioux Falls, SD, USA

## Abstract

**Background:**

Epstein-Barr virus- (EBV-) associated lymphoproliferative disease (LPD) is a rare condition, usually occurring in immunocompromised patients. We report a case of EBV-associated LPD in a patient with severe celiac disease, the first report to describe this syndrome in a patient with this diagnosis.

**Case Summary:**

A 69-year-old Caucasian woman with recent diagnosis of celiac sprue presented to our hospital with persistent diarrhea, abdominal pain, weight loss, and fatigue despite adherence to gluten-free diet for a number of weeks prior to presentation. She underwent evaluation for occult malignancy and was found to have diffuse intra-abdominal mesenteric lymphadenopathy on CT scan. Biopsy of mesenteric nodes revealed an EBV positive, CD20 positive mixed lymphoproliferative process with T-cell predominance, but without a monoclonal cell population felt to be consistent with EBV-associated LPD. Bone marrow biopsy revealed hemophagocytic lymphohistiocytosis, complicating her course. She was treated with steroids and rituximab but continued to decline, eventually developing MSSA bacteremia and succumbing to her disease.

**Conclusion:**

To our knowledge, this is the first report of the constellation of celiac sprue, EBV-associated LPD, and hemophagocytic lymphohistiocytosis. Providers caring for patients with severe, uncontrolled celiac disease and adenopathy should consider EBV-associated LPD.

## 1. Introduction

Epstein-Barr virus (EBV), also called human herpes virus 4 (HHV-4), is one of the eight known human herpes viruses [1]. It was discovered in 1964 by electron microscopy of African Burkitt lymphoma cells [2]. Antibodies to EBV have been demonstrated in all population groups worldwide; approximately 90 percent of adults worldwide are EBV-seropositive [3].

The majority of EBV infections are subclinical [3]. Infectious mononucleosis is the most common clinical manifestation of EBV infection [4]. Epstein-Barr virus (EBV) has been implicated in the pathogenesis of B-cell lymphomas, T-cell lymphomas, Hodgkin lymphoma, and nasopharyngeal carcinomas and has associated with the development of EBV-associated lymphoproliferative disorders (LPD) as well. EBV-associated LPD occur in a spectrum, ranging from polyclonal B-cell proliferation to clonal, malignant B-cell lymphoma and most commonly occur in immunocompromised patients, especially in posttransplant and HIV-positive patients. [3, 5, 6] EBV-associated LPD are increasingly being recognized in the elderly population as well, likely due to age-related immunosenescence [7]. However, the constellation of celiac sprue, EBV-associated LPD, and hemophagocytic lymphohistiocytosis is unique in the literature.

## 2. Case Report

A 69-year-old Caucasian female with recently diagnosed celiac sprue presented with fever, nausea, abdominal pain, diarrhea, and weight loss despite adherence to a gluten-free diet. Past medical history included hypothyroidism, hypertension, and atrial fibrillation. She initially presented to an outside facility several months prior to presentation to our facility where she had a small bowel biopsy showing villous blunting consistent with celiac sprue as well as positive autoantibodies for gliadin and tissue transglutaminase. She was started on a gluten-free diet and discharged. Despite adhering to the gluten-free diet, she lost approximately 15 pounds over the month prior to presentation at our facility.

Given persistent symptoms, she was admitted to our institution, where initial blood work noted pancytopenia, elevated serum ferritin, elevated serum triglycerides, and low serum fibrinogen, raising clinical concern for hemophagocytic lymphohistiocytosis (HLH). Computed tomography (CT) of the chest, abdomen, and pelvis noted extensive lymphadenopathy throughout the abdomen with the largest nodes including a 2.9 cm mesenteric node in the left abdomen, a 2.4 cm node near the left iliac crest, and a 3.4 cm mass adjacent to the cecum.

**Figure 1:**
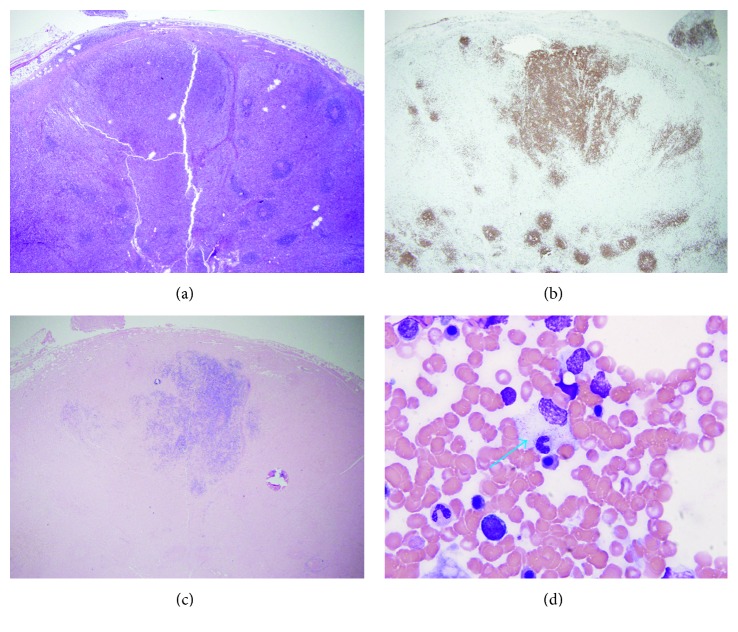
(a) Hematoxylin and eosin (H&E) stained mesenteric lymph node reveals effacement of nodal architecture by paracortical expansion of an atypical lymphoid population. Scattered small lymphoid follicles are noted (2x). (b) A CD20 immunohistochemical stain highlights sheets of monotonous, medium-sized cells, identifying the cells as B-lymphocytes (2x). (c) EBER, a nuclear stain for EBV-encoded RNA, is expressed by the B-lymphocyte population and scattered throughout the paracortical expansion, suggesting an EBV-related lymphoproliferative disorder (2x). (d) H&E staining of the bone marrow aspirate shows a hemophagocytic histiocyte (arrow), which contains a leukocyte and appears to be in the process of engulfing a red blood cell (100x).

A needle biopsy of an intra-abdominal lymph node demonstrated a lymphoproliferative process, with a predominance of CD4-positive T-lymphocytes. Following needle biopsy, an excisional biopsy was obtained revealing a polymorphic-type EBV-positive lymphoproliferation with extensive paracortical T-cell hyperplasia. The paracortical expansion was comprised of predominantly CD4-positive polymorphic T cells that stained positively for CD2, CD3, and CD5, with partial loss of CD7 (Figure 1(a)). A monotonous population of CD20-positive B cells was also identified that coexpress MUM-1 and was positive for EBER (Epstein-Barr virus encoded messenger RNA) (Figures 1(b) and 1(c)). T-cell receptor gene rearrangement studies by PCR were not performed; however, gene rearrangement studies for TCR-gamma/delta and TCR-betaF1 by immunohistochemistry were performed. TCR-betaF1 was positive in the polymorphic T-cell population within the paracortical expansion. TCR-gamma/delta stained a small subset of T cells. The final diagnosis was a CD20-positive, EBV-positive lymphoproliferative disorder, which did not meet criteria for frank lymphoma and was not monoclonal in nature.

Following the diagnosis of EBV-associated LPD, peripheral blood EBV DNA quantitation via PCR was markedly positive at 9367 copies/mL (<200 copies/mL); to our knowledge, the patient's EBV status was unknown until this point. Subsequent bone marrow biopsy was performed to further evaluate her pancytopenia. This revealed marked hemophagocytic changes consistent with HLH (Figure 1(d)). She was started on a course of rituximab and methylprednisolone for EBV-associated LPD with HLH. She subsequently developed methicillin-sensitive S*taphylococcus aureus (MSSA)* bacteremia, further worsening her clinical status. Following development of bacteremia, the patient and her family decided to pursue comfort care, and she succumbed while receiving comfort cares 25 days after initial presentation to our hospital.

## 3. Discussion

We describe a case of EBV-associated LPD, a rare disorder which typically occurs in immunocompromised patients and has been most commonly described in posttransplant and HIV-positive patients. To our knowledge, this is the first case report to describe EBV-associated LPD disorder associated with celiac disease.

Celiac disease has been associated with many different subtypes of lymphoma [8, 9], although lymphoma remains an uncommon complication in this population. Enteropathy-associated T-cell lymphoma (EATL) is a distinct lymphoma subtype highly associated with celiac disease, although B-cell lymphoma in association with celiac disease has also been described [10]. Patients with celiac-associated B cell lymphoma may have a better prognosis than those with EATL [11].

To our knowledge, EBV-associated LPD has not been described in a patient with celiac disease, although EBV has been implicated in some cases of EATL [10] and active EBV has been detected in small bowel biopsies in up to 70% of patients with refractory celiac disease [12]. In this case, the strength of the link between the diagnosis of celiac disease and the EBV-associated LPD is somewhat difficult to discern, as the patient also had multiple other factors potentially causing relative immunosuppression including malnutrition secondary to celiac disease as well as age-related immunosenescence, but this case should alert clinicians to the possibility of EBV-associated LPD when presented with a patient with celiac disease and associated adenopathy concerning for lymphoma.

In patients with early, nonclonal EBV-associated LPD on immunosuppression either following solid organ transplant or due to a chronic autoimmune condition, modulation of immunosuppression is the initial management recommendation. Modulation of immunosuppressive therapy induces tumor regression in 25%–50% of patients [13–15]. In patients with a polyclonal EBV-associated LPD, rituximab is often added to modulation of immunosuppression [16, 17], as rituximab is generally well tolerated and rapidly depletes mature B-lymphocytes, the major reservoir of EBV [18]. In patients with monoclonal EBV-associated LPD or disease refractory to rituximab and modulation of immunosuppression, rituximab is typically combined with chemotherapy directed at the particular lymphoma subtype described [17]. In this case, given her frail state at diagnosis and patient hesitance regarding more aggressive therapy, treatment with rituximab was combined with high dose steroids as opposed to multiagent chemotherapy. Unfortunately, the patient developed a significant infectious complication and succumbed to her disease prior to an opportunity to assess her response to the above therapy.

Another confounding feature of this case is the concurrent diagnosis of HLH. HLH has been well described in concert with both B-cell lymphomas [19] and EBV infection [20] as a trigger for HLH. Our patient met 5/8 of the diagnostic criteria for HLH (fever, pancytopenia, hypertriglyceridemia, elevated serum ferritin, and hemophagocytosis on histopathological examination of bone marrow) [21]. Serum soluble IL2 receptor level was not checked as our patient already met the diagnostic criteria for HLH. The treatment regimen of HLH is chemoimmunotherapy based with the most commonly used regimen being etoposide, cyclosporin A, and dexamethasone [22]. Rituximab is also commonly utilized in the treatment of EBV-associated HLH [21].

In conclusion, we represent a case of EBV-associated LPD occurring in a patient with refractory celiac disease complicated by hemophagocytic lymphohistiocytosis. This case demonstrates the difficulty in diagnosis and management of EBV-associated LPD and will hopefully lead to increased awareness of this possible diagnosis amongst clinicians treating patients with refractory celiac disease.
